# Effect of rye bread breakfasts on subjective hunger and satiety: a randomized controlled trial

**DOI:** 10.1186/1475-2891-8-39

**Published:** 2009-08-26

**Authors:** Hanna Isaksson, Helena Fredriksson, Roger Andersson, Johan Olsson, Per Åman

**Affiliations:** 1 Department of Food Science, Swedish University of Agricultural Sciences, SE-750 07 Uppsala, Sweden; 2 Lantmännen R&D, St Göransgatan 160 A, SE-104 25 Stockholm, Sweden; 3 KPL Good-Food-Practice AB, Dag Hammarskjölds väg 10B, SE-751 83 Uppsala, Sweden

## Abstract

**Background:**

Several studies report that dietary fibre from different sources promotes the feeling of satiety and suppresses hunger. However, results for cereal fibre from rye are essentially lacking. The aim of the present study was to investigate subjective appetite during 8 h after intake of iso-caloric rye bread breakfasts varying in rye dietary fibre composition and content.

**Methods:**

The study was divided into two parts. The first part (n = 16) compared the satiating effect of iso-caloric bread breakfasts including different milling fractions of rye (bran, intermediate fraction (B4) and sifted flour). The second part (n = 16) investigated the dose-response effect of rye bran and intermediate rye fraction, each providing 5 or 8 g of dietary fibre per iso-caloric bread breakfast. Both study parts used a wheat bread breakfast as reference and a randomised, within-subject comparison design. Appetite (hunger, satiety and desire to eat) was rated regularly from just before breakfast at 08:00 until 16:00. Amount, type and timing of food and drink intake were standardised during the study period.

**Results:**

The Milling fractions study showed that each of the rye breakfasts resulted in a suppressed appetite during the time period before lunch (08:30-12:00) compared with the wheat reference bread breakfast. At a comparison between the rye bread breakfasts the one with rye bran induced the strongest effect on satiety. In the afternoon the effect from all three rye bread breakfasts could still be seen as a decreased hunger and desire to eat compared to the wheat reference bread breakfast.

In the Dose-response study both levels of rye bran and the lower level of intermediate rye fraction resulted in an increased satiety before lunch compared with the wheat reference bread breakfast. Neither the variation in composition between the milling fractions nor the different doses resulted in significant differences in any of the appetite ratings when compared with one another.

**Conclusion:**

The results show that rye bread can be used to decrease hunger feelings both before and after lunch when included in a breakfast meal. Rye bran induces a stronger effect on satiety than the other two rye fractions used when served in iso-caloric portions.

**Trial Registration:**

Trial registration number NCT00876785

## Background

Energy-dense foods that require little effort to consume and that are rapidly digested may cause passive over-consumption by failure to provide a feeling of fullness corresponding to the energy content. A diet that is predominantly based on such foods may lead to overweight. It is therefore important to identify properties of foods that facilitate energy balance by creating a high satiety per calorie. A number of studies have confirmed that foods naturally rich in dietary fibre promote the feeling of fullness and reduce hunger in the short term [1]. A recent review [2] concluded that a diet rich in whole grain cereals was associated with a lower body mass index and a lower risk for overweight. In the Western world the majority of the whole grain products eaten are based on wheat, while the consumption of oats and especially rye and barley is much lower. Oats are mainly consumed in Northern Europe, Northern America and Australia, whereas rye consumption is essentially limited to the Northern, Central and Eastern Europe [3]. Wheat is consequently the most studied whole grain cereal in relation to health status including satiety and weight regulation. Very few studies have compared the satiating capacity of dietary fibre from different types of cereal grains or cereal fractions. One study showed that rye consumed as whole boiled kernels resulted in higher ratings of subjective satiety compared with wheat kernels [4]. Results from a smaller investigation [5] indicated that whole grain rye is more satiating than whole grain oats, when eaten as porridge made from rolled flakes. That rye possibly has superior satiating properties may be due to its high dietary fibre content and possibly fibre composition. The main dietary fibre components of whole grain rye are arabinoxylan (AX) (8.0-12%), fructan (4.6-6.6%), β-glucan (1.3-2.2%) and cellulose (1.0-1.7%) [3]. The AX structure varies within different parts of the grain and a higher degree of water-extractable AX is found in the endosperm and a lower degree in the outer parts of the grain. Rye has been shown to elicit a lower rise in post-prandial insulin compared with wheat, despite a similar rise in blood glucose [6]. The relatively low insulin response was evident for bread with intact grains of rye [7] as well as for bread made with whole grain rye flour [8]. The insulin response does not seem to be related to any specific part of the rye grain but has been shown to occur after consumption of bran as well as sifted flour [9]. This difference in metabolic response between rye and wheat may also affect satiety.

In the present study we investigated subjective appetite (hunger, satiety and desire to eat) during 8 h after consumption of iso-caloric rye bread breakfasts. The study was performed in two parts. The first part (Milling fractions study) compared different milling fractions of rye. The second part (Dose-response study) included different levels of dietary fibre from rye.

## Materials and methods

### Participants

The criteria for inclusion were the following: age between 20 and 60 years; body mass index (BMI) 18-27 kg/m^2^; a habit of consuming breakfast, lunch and dinner everyday; fasting plasma glucose 4.0-6.1 mmol/L; haemoglobin (Hb) in men 130-170 g/L, in women 120-150 g/L; alaninaminotransferase (ALT) in men 0.15-1.1 μkat/L, in women 0.15-0.75 μkat/L; thyroid-stimulating hormone (TSH) 0.3-4.0 mlE/L and willingness to comply with the study procedures. Exclusion criteria were the following: intake of medicine likely to affect appetite or food intake; any medical condition involving the gastrointestinal tract; eating disorder; smoking; consumption of more than three cups of coffee per day; change in body weight of more than 10% three months prior to screening; consumption of any restricted diet such as vegan, gluten-free, slimming etc.; and pregnancy, lactation or wish to become pregnant during the study period. Information about the study was first sent out by e-mail, to employees at Lantmännen, Järna, Sweden. Potential participants were screened in a telephone interview and undertook a health control before being recruited. A total of eleven participants is the minimum number to detect a difference of 10 mm in hunger ratings over 4.5 h [10]. Smaller differences than this may be irrelevant in a real life and we chose not to power the study to detect differences much below 10 mm. In all, 16 participants, 14 female and 2 male, met the criteria and were included in the Milling fractions study (table 1). A different group of 19 participants, 15 female and 4 male, were recruited to the Dose-response study.

**Table 1:** Participant characteristics

	Milling fractions study^1^		Dose-response study^2^	
		
	Mean ± SD	Range	Mean ± SD	Range
Age (years)	35 ± 10	24-59	38 ± 12	23-60
Body mass index (kg/m^2^)	22 ± 2.8	18-27	23 ± 2.0	19-26
Capillary fasting plasma glucose (mmol/L)	5.1 ± 0.5	4.1-5.9	5.4 ± 0.4	4.6-6.1
Thyroid-stimulating hormone (mlE/L)	1.6 ± 0.7	0.9-3.8	1.3 ± 0.6	0.3-2.6
Haemoglobin (g/L)	136 ± 6.0	125-148	142 ± 9.0	127-156
Alaninaminotransferase (μkat/L)	0.3 ± 0.1	0.2-0.7	0.6 ± 0.2	0.3-1.1

^1 ^n = 16; 14 female, 2 male

^2 ^n = 16; 13 female, 3 male

### Study design

A randomised, crossover design was used to compare the effects on subjective appetite 8 h after consumption of iso-caloric breakfast meals. The aim of the study and the content of the test products were not known to the participants. Each participant acted as his/her own control and received each of the bread breakfasts in a randomised order on different occasions, separated by six to eight days. In the time between the test days, the participants kept their ordinary diet. On the day prior to each test day, participants were instructed not to conduct any vigorous physical activity or drink any alcoholic beverages; not to eat or drink after 20:00 and to eat similar type and amount of evening meal. During the test day the participants were asked to fill out a diary, briefly noting their food and beverage consumption and exercise patterns from the previous evening, to ensure that instructions had been followed. Upon arrival on the morning of the test days (08:00) the participants were served one of the breads together with additional breakfast foods, identical on all test occasions in both parts of the study. During intake of the breakfast, the participants were seated in individual booths and instructed not to talk to one another. At 12:00 the participants were given a standardised lunch meal. The breakfast and lunch meals had to be consumed entirely within 30 min. At 14:00 they were allowed to drink coffee or tea. The hot drink, along with a voluntary choice of milk and sugar, was kept identical for each of the participants on all test occasions. Subjective feelings of appetite (hunger, satiety and desire to eat) were assessed every half hour, starting at 08:00 and continuing until 16:00. The first recording was made in the fasted state immediately before breakfast at 08:00. The data were collected using a specially designed programme [11] on a palm computer (z22, China). At each appetite recording an alarm went off to remind the participant and these three questions were presented in sequence: 'How hungry do you feel right now?'; 'How full do you feel right now?' and 'How strong is your desire to eat right now?', along with three respective scales marked at opposite ends: not at all hungry/extremely hungry, not at all full/extremely full, extremely strong/not at all strong. The computer mimics the use of pen and paper as it is operated by tapping on the screen with a rubber pen. Like the conventional 100 mm visual analogue scales (VAS) [12], the computerised system translates the mark that the participant makes along the scale to a number between 0 and 100. Written informed consent was obtained from each participant. The study was carried out in compliance with the Helsinki Declaration and approved by the Ethics Committee at Uppsala University.

### Rye and wheat material

Three rye milling fractions were used (table 2): rye bran (20% of the total grain), an intermediate rye fraction (B4) taken from the fourth brake roll in the milling process and sifted rye flour (80% of the total grain). The rye bran was high in dietary fibre, dark brown in colour and had a distinct taste of rye. The intermediate rye fraction, with a lower content of dietary fibre, was lighter in colour and much milder in flavour as the outermost part of the grain was absent. This fraction was chosen after a screening of ten milling fractions (analysed at Nordmills, Malmö, Sweden) because of its relatively high content of extractable dietary fibre. The sifted rye flour had the lowest content of dietary fibre of the three, was lightest in colour and had an even milder flavour. After the milling process, the rye bran contained larger particles than the intermediate rye fraction and sifted rye flour. To reduce the possible effects of structure/particle size, the rye bran was milled to a fine flour, similar to that of the intermediate rye fraction and sifted rye flour. Sifted wheat flour of high quality (Bagerivetemjöl, Nordmills, Malmö, Sweden) was used in all breads.

**Table 2:** Nutritional composition of the rye and wheat milling fractions used in the breads (per 100 g)^1^

	Rye			Wheat
		
	Bran	Intermediate fraction	Sifted flour	Sifted flour
Energy kJ (kcal)	1000 (240)	1250 (300)	1370 (330)	1430 (340)
Water (g)	10.5	12.2	12.9	13.8
Protein (g)	16	13	8.1	11
Fat (g)	4.4	2.9	1.7	2.0
Available carbohydrate (g)	33	54	69	68
Ash (g)	3.6	1.4	0.8	0.6
Total dietary fibre (g)	32.2	16.4	8.0	2.5
extractable (g)	5.2	7.2	3.5	0.7
unextractable (g)	27	9.2	4.5	1.8

^1^The samples were analysed in duplicate by AnalyCen, Lidköping, Sweden. Total dietary fibre was analysed according to AOAC 45.4.07 and unextractable dietary fibre according to AOAC 32.1.16. Extractable dietary fibre was calculated by difference. Available carbohydrate was calculated by difference (total weight minus water, protein, fat, ash and total dietary fibre).

### Test breads

To create breads with acceptable palatability and relatively soft texture, the amount of rye that could be used was limited to 60% of the total amount of flour for rye bran and 75% for the intermediate rye fraction. A higher content of rye resulted in compact breads with low ability to rise during fermentation.

When baking each type of bread, the amounts of ingredients (table 3) were scaled up. Rye and wheat flour were mixed with gluten and salt, after which rape seed oil, syrup, yeast and water (25°C) were added. The dough was kneaded for 7 min using a kitchen food processor (Varimixer, Bjorn, Wodschow & Co, Denmark) and then left to rise for 40 min (34°C). Thereafter the dough was divided into pieces corresponding to the portion sizes used and left to rise for another 40 min (rye bran and intermediate rye fraction breads) or 25 min (the sifted rye flour bread and wheat reference bread). The breads were baked at a temperature of 200°C for 10 min. After cooling for approximately one hour the breads were stored frozen until the night before each test breakfast.

**Table 3:** Cereal ingredients (g) and water (g) used in one bread portion^1 ^(1090 kJ^2^/260 kcal)

	Rye			Wheat	Water^4^
			
	Bran	Intermediate Fraction	Sifted flour	Sifted flour ^3^	
Milling fractions study^5^					

Rye bran bread	42	-	-	30	80
Intermediate rye fraction bread	-	34	-	30	70
Sifted rye flour bread	-	-	31	30	45
Wheat reference bread	-	-	-	60	40

Dose-response study^6^					

Rye bran bread (8 g dietary fibre)	25	-	-	42	60
Rye bran bread (5 g dietary fibre)	16	-	-	49	55
Intermediate rye fraction bread (8 g dietary fibre)	-	49	-	17	70
Intermediate rye fraction bread (5 g dietary fibre)	-	30	-	33	70
Wheat reference bread	-	-	-	60	40

^1^Additional ingredients used for each bread portion were: 3 g syrup, 3 g rape seed oil, 4 g gluten, 4 g yeast and 1 g salt.

^2^The energy value of the breads were calculated based on the energy values of the ingredients using Dietist XP, version 3.0, Bromma, Sweden.

^3 ^The amount of wheat flour was adjusted to equalise the amount of energy (kJ) per bread portion.

^4 ^The water content was adjusted to create a good dough consistency. More water was required at higher levels of dietary fibre, especially extractable.

^5 ^The amount of rye corresponded to 240 kJ (100 kcal) per portion.

^6^The amount of rye corresponded to the two levels of dietary fibre.

The different types of breads contained the same amount of energy per portion and were similar in protein, fat and available carbohydrate composition (table 4). The main difference was dietary fibre content and subsequently the weight of each portion.

**Table 4:** Nutrient content per bread portion. One portion equals 1090 kJ (260 kcal)^1^

	Portion size (g)	Protein (g)	Fat (g)	Available carbohydrates (g)	Total dietary fibre (g)	Dietary fibre from rye (g)
Milling fractions study						

Rye bran bread	133	13.5	5.5	38	14.5	13.6
Intermediate rye fraction bread	120	11.0	4.5	42	6.5	5.6
Sifted rye flour bread	100	9.5	4.0	45	3.5	2.5
Wheat reference bread	98	10.0	4.0	44	1.5	0

Dose-response study						

Bran bread (8 g dietary fibre)	121	12.0	5.0	40	9.0	8.0
Bran bread (5 g dietary fibre)	114	11.5	4.5	42	6.0	5.0
Intermediate fraction bread (8 g dietary fibre)	126	11.5	4.5	41	8.5	8.0
Intermediate fraction bread (5 g dietary fibre)	123	11.0	4.5	42	6.0	5.0
Wheat reference bread	98	10.0	4.0	44	1.5	0

^1^Nutrient and energy content were calculated based on the ingredients included (Dietist XP, version 3.0, Bromma, Sweden). The composition of the rye and wheat flours was analysed by AnalyCen, Lidköping, Sweden) and nutrient composition for additional ingredients were manufacturer's values. Thus, any alteration in composition that occurred during the baking process was not taken into account.

### Meals

Food intake was standardised in terms of type, amount and timing during the test day. The rye breads and the wheat reference bread were served in random order on separate occasions as breakfast meals with identical additional foods: 10 g of margarine (40% fat), 25 g of apricot marmalade, 15 g cheese (26% fat), 200 g of milk (0.5% fat) and one cup of tea or coffee. The participants were allowed to choose between coffee and tea on the first test day and then received the same type of drink for the following test days. The breakfast meal provided in total 1960 kJ (470 kcal). Although dietary fibre is known to contribute energy from short-chain fatty acids following colonic fermentation at around 8.4 kJ/g (2 kcal) [13], there is no universally agreed value and for this reason it was not included in the energy values given for the cereal flour. The standardised lunch consisted of a ready-made vegetarian pasta dish (Pasta pomodoro e mozarella, Gooh!, Stockholm, Sweden) (400 g, 2040 kJ/480 kcal, 21 g protein, 64 g carbohydrates, 16 g fat), 50 g of cocktail tomatoes and 50 g of cucumber. At 14:00 the participants had a banana (Milling fractions study) or an apple (Dose-response study) and could choose to drink a cup of tea or coffee, the drink was then being kept identical on the following test days.

### Data analysis

Ratings for satiety, hunger and desire to eat were analysed using Minitab (version 15, LEAD Technologies, Inc, USA). The level of significance was set at p < 0.05. ANOVA was performed as paired t-tests and Tukey comparisons using participants as random effect and type of breakfast and time points as fixed effects. Separate analysis was done for the morning (08:30-12:00) and afternoon ratings (12:30-16:00).

## Results

All participants finished the breakfast and lunch meals completely according to instructions. No adverse events were recorded and no one had problems finishing the test meals well within 30 min.

Before breakfast the mean ratings for hunger, satiety or desire to eat were similar between test days. Appetite ratings showed a clear effect of time after breakfast and after lunch, i.e. the VAS ratings visibly demonstrated that the participants responded with lessened hunger and increased satiety directly after the meals and then consecutively rated less satiety and stronger hunger as the time for the next meal approached.

### Milling fractions study

The Milling fractions study was designed to compare the satiating capacity of iso-caloric portions of three rye milling fractions included in bread breakfasts. Sifted wheat bread breakfast was used as reference. All of the 16 participants recruited initially (table 1) complied with the study procedures and completed the study.

**Figure 1:**
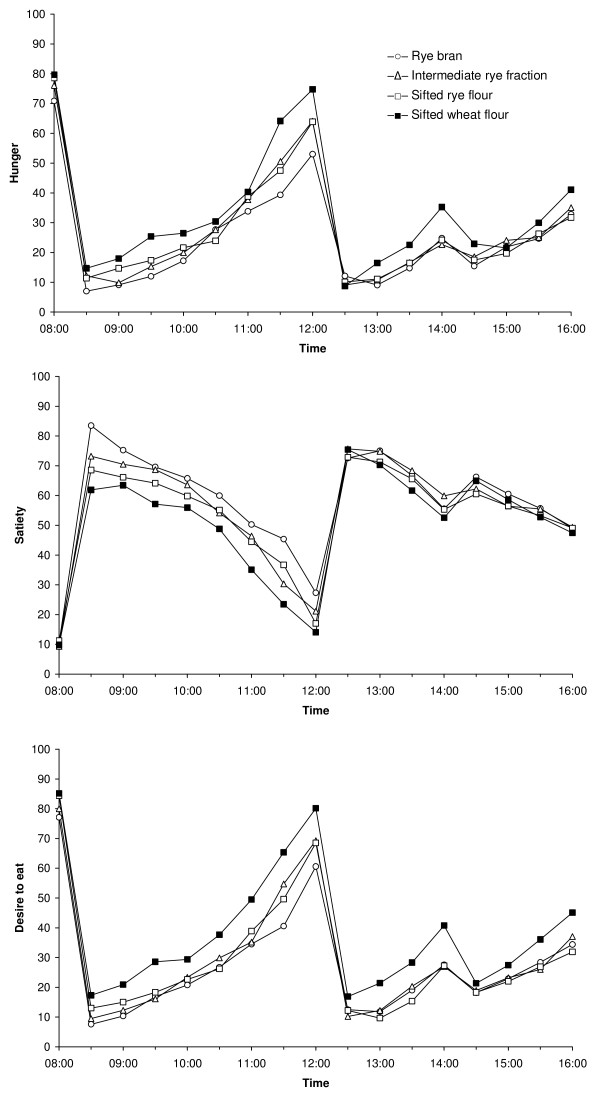
**Milling fractions study**. Mean appetite ratings (n = 16) after consumption of the four breakfast meals including breads with rye and wheat milling fractions.

In the morning (08:30-12:00) the rye bran bread breakfast induced the strongest effect on satiety, stronger than that of the intermediate rye fraction and sifted rye flour bread breakfasts (figure 1, table 5). Further, each of the three rye bread breakfasts resulted in an increased satiety, decreased hunger and decreased desire to eat compared to the wheat reference bread breakfast.

**Table 5:** Statistical evaluation of appetite ratings (n = 16) for time intervals following bread breakfasts (Milling fractions study)^1^

	Time intervals	
	
	08:30-12:00	12:30-16:00
	
	Hunger	
Rye bran bread	a	a
Intermediate rye fraction bread	a	a
Sifted rye flour bread	a	a
Wheat reference bread	c	c
	**Satiety**	

Rye bran bread	a	a
Intermediate rye fraction bread	b	a
Sifted rye flour bread	b	a
Wheat reference bread	c	a
	**Desire to eat**	

Rye bran bread	a	a
Intermediate rye fraction bread	a	a
Sifted rye flour bread	a	a
Wheat reference bread	c	c

^1^Different letters within columns indicate significant difference (p < 0.05).

In the afternoon (12:30-16:00), after the standardised lunch, type of breakfast bread did not affect satiety. However, hunger and desire to eat was lower after consumption of each of the three rye bread breakfasts compared with the wheat reference bread breakfast. When the three rye bread breakfasts were compared with each other, no significant differences were seen in any of the appetite measures during the afternoon.

### Dose-response study

The Dose-response study was designed to investigate the satiating capacity of four rye bread breakfasts with rye bran and intermediate rye fraction, each in amounts providing 5 or 8 g of rye dietary fibre/portion. Of the 19 participants recruited initially, 16 completed the study (table 1). The three exclusions were due to failure to comply with the study procedures.

**Figure 2:**
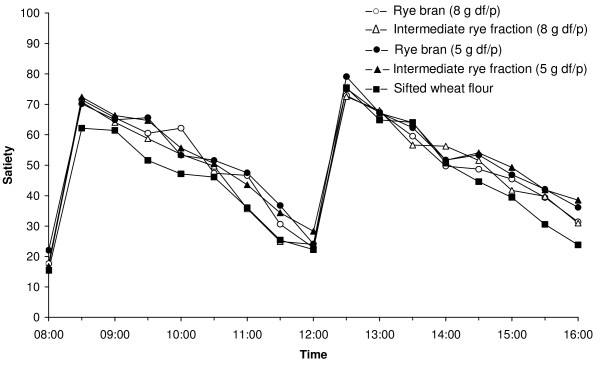
**Dose-response study**. Mean satiety ratings (n = 16) after consumption of the five breakfast meals including breads with rye and wheat milling fractions (df/p refers to dietary fibre/portion).

On comparisons with the wheat reference bread breakfast the results demonstrated a significantly increased satiety (08:30-12:00) even at the lower levels of rye bran and intermediate rye fraction, as well as for the rye bran bread breakfast, that provided 8 g of rye dietary fibre/portion (figure 2). The intermediate rye fraction breakfast, which provided 8 g of rye dietary fibre/portion, did not significantly increase satiety. No effects were seen on hunger or desire to eat (data not shown).

In the afternoon (12:30-16:00), after the standardised lunch, type of breakfast bread did not significantly affect appetite. No significant differences were seen in any of the appetite measures, when the four rye bread breakfasts were compared with each other. However, in the late afternoon there was tendency for increased satiety for all the rye bread breakfasts compared to the wheat reference breakfast.

## Discussion

The aim of this study was to investigate subjective appetite during 8 h after intake of iso-caloric rye bread breakfasts. Finely milled rye fractions were compared at different levels. When the appetite ratings after intake of the rye bread breakfasts were compared with those after consumption of the wheat reference bread breakfast, significant effects on appetite were apparent in both studies. At comparison between the three milling fractions, a distinct effect on satiety was seen after the breakfast with rye bran, followed by the other two rye bread breakfasts. In the second part no differences between the rye breads were found, despite differences in the amount and composition of rye. This may be explained by some property present in all of the rye breads, for instance relating to bread structure [9].

The levels of rye used in the breads were based on realistic amounts to create palatable, voluminous bread. The bread portion, together with additional breakfast foods, comprised what would be considered a normal breakfast meal. The amount of calories corresponded to recommended breakfast intake. These considerations somewhat limited the amount of rye that could be used in the breakfast meals. Nevertheless, in both parts of the study the rye breads had an affect on appetite compared with the wheat reference bread indicating that the method was sufficiently sensitive.

In an earlier study [14] with similar design we showed that a rye breakfast (porridge made from whole grain rye flakes) was followed by an increased satiety not only in the morning but also in the afternoon, following a standardised lunch. When designing the present study we were interested in this effect, hence the standardised lunch and continued appetite assessment in the afternoon. The results were not as clear as in the previous study, but a reduced hunger and desire to eat in the afternoon was evident for all the rye bread breakfasts in the Milling fractions study. The effect may in part be related to processing and food preparation, since in the previous study rye flakes were used to make the porridge whereas in the present study finely milled flour was baked into breads.

The mechanisms underlying the satiating effects were not investigated in the present study. However, the satiating properties of dietary fibre have been related to several stages in the physiological processes of short-term appetite regulation [15]. These include bulking effects resulting in increased extension of the stomach and, for some viscous dietary fibre, delayed gastric empting causing the early signals of satiation to increase. Furthermore, pre-absorptive hormonal signalling at the level of the small intestine is essential in the induction and maintenance of satiety. Dietary fibre that delay absorption of nutrients may therefore lead to prolonged satiety by increasing the time that macronutrients are in contact with the absorptive surfaces. Finally, end products caused by colonic fermentation of dietary fibre, such as acetate and propionate, have been suggested to affect satiety [16]. How this effect is mediated is not clear. Suggested mechanisms are stimulated release of satiety hormones (GLP-1, PYY) by L-cells in the colon [16]. The increased satiety during the afternoon, several hours after the test breakfast, may be explained by colonic events.

In a broader perspective it is important to consider whether a dietary pattern providing a high satiety per calorie is potent enough to affect energy intake in real life, and in the long run facilitate weight maintenance and/or weight loss. It is evident that satiety signals are being overwhelmed to a certain degree by the potency of external cues to eating [17]. Longer term experimental studies show that an increased intake of dietary fibre results in a spontaneously lowered energy intake and a loss of body weight [18]. These results from experimental studies are also supported by observational studies that a diet low in dietary fibre is associated with an increased risk of obesity [19,20].

The present study provides support for altering appetite towards an increased satiety for up to 8 h by including rye breads in a breakfast meal.

## Competing interests

The study was financed by the producer of the test products, Lantmännen. HI and HF are employed by Lantmännen R&D. The authors declare that the data presented in this publication represent a complete, true and faithful representation of the work performed.

## Authors' contributions

HI, HF, RA, PÅ and JO participated in designing the study. HI recruited the participants and performed the study. RA and HI analysed the data. HI wrote the manuscript. All authors participated in revising the manuscript and read and approved the final manuscript.

## References

[B1] Slavin J Green H Dietary fibre and satiety Nutr Bull 2007 32 suppl 1 32 42 10.1111/j.1467-3010.2007.00603.x

[B2] Williams PG Grafenauer SJ O'Shea JE Cereal grains, legumes, and weight management: a comprehensive review of the scientific evidence Nutr Rev 2008 66 171 182 10.1111/j.1753-4887.2008.00022.x 18366531

[B3] Kamal-Eldin A Åman P Zhang J-X Bach Knudsen K-E Poutanen K Hamaker BR Rye bread and other rye products Technology of functional cereal products 2007 Cambridge: Woodhead Publishing Limited 233 260

[B4] Östman E Silva LB Björck I Influence of food form on acute and second-meal glucose tolerance to wheat and rye products Poster presented at International ICC Conference on Rye 2007 http://www.appliednutrition.lth.se/forskning/postrar/

[B5] Isaksson H Fredriksson F Åman P Tetens I The effect of cereal based breakfast meals on satiety and voluntary energy intake Proceedings of the Nordic Nutrition Conference: 1-4 June 2008; Copenhagen 2008 69

[B6] Leinonen K Liukkonen K Poutanen K Uusitupa M Mykkänen H Rye bread decreases postprandial insulin response but does not alter glucose response in healthy Finnish subjects Eur J Clin Nutr 1999 53 262 267 10.1038/sj.ejcn.1600716 10334650

[B7] Liljeberg H Granfeldt Y Björck I Metabolic responses to starch in bread containing intact kernels versus milled flour Eur J Clin Nutr 1992 46 561 575 1396475

[B8] Juntunen KS Niskanen LK Liukkonen KH Poutanen KS Holst JJ Mykkänen HM Postprandial glucose, insulin, and incretin responses to grain products in healthy subjects Am J Clin Nutr 2002 75 254 262 11815315 10.1093/ajcn/75.2.254

[B9] Juntunen KS Laaksonen DE Autio K Niskanen LK Holst JJ Savolainen KE Liukkonen KH Poutanen KS Mykkänen HM Structural differences between rye and wheat breads but not total fiber content may explain the lower postprandial insulin response to rye bread Am J Clin Nutr 2003 78 957 964 14594782 10.1093/ajcn/78.5.957

[B10] Flint A Raben A Blundell JE Astrup A Reproducibility, power and validity of visual analogue scales in assessment of appetite sensations in single test meal studies Int J Obes Relat Metab Disord 2000 24 38 48 10.1038/sj.ijo.0801083 10702749

[B11] Stratton RJ Stubbs RJ Hughes DA King N Blundell JE Elia M Comparison of the traditional paper visual analogue scales questionnaires with an Apple Newton electronic rating system (EARS) in free living subjects feeding ad libitum Eur J of Clin Nutr 1998 52 737 741 10.1038/sj.ejcn.1600636 9805221

[B12] Stubbs RJ Hughes DA Johnstone AM Rowley E Reid C Elia M Stratton R Delargy H King N Blundell JE The use of visual analogue scales to assess motivation to eat in human subjects: a review of their reliability and validity with an evaluation of new hand-held computerized systems for temporal tracking of appetite ratings Br J Nutr 2000 84 405 415 10.1017/S0007114500001719 11103211

[B13] FAO Food and Nutrition Paper No. 66 Carbohydrates in human nutrition. Report of a Joint FAO/WHO Expert Consultation Rome 1998 9743703

[B14] Isaksson H Sundberg B Åman P Fredriksson H Olsson J Whole grain rye porridge breakfast improves satiety compared to refined wheat bread breakfast Food Nutr Res [Online] 2008 52 0 10.3402/fnr.v52i0.1809 PMC2596730 19109656

[B15] Burton-Freeman B Dietary fibre and energy regulation J Nutr 2000 130 Suppl 2 272 275 10.1038/oby.2006.298 10721886

[B16] Peters HPF Mela DJ Harris RBS, Mattes RD The role of the gastrointestinal tract in satiation, satiety, and, food intake: evidence from research in humans Appetite and food intake: behavioural and physiological considerations 2008 Boca Raton: Taylor & Francis 187 211

[B17] Blundell JE Perspective on the central control of appetite Obesity 2006 14 Suppl 4 160 163 10.1038/oby.2006.298 16931499

[B18] Howarth NC Saltzman E Roberts SB Dietary fibre and weight regulation Nutr Rev 2001 59 129 139 11396693 10.1111/j.1753-4887.2001.tb07001.x

[B19] WHO Technical Report Series No 916 Diet, Nutrition and the Prevention of Chronic Diseases Geneva 2003 12768890

[B20] Lairon D Dietary fiber intake and risk factors for cardiovascular disease in French adults Am J Clin Nutr 2005 82 1185 1194 16332650 10.1093/ajcn/82.6.1185

